# An Experiment in the Transmission of Syphilis to Calves1Read before the Pathological Society of Philadelphia, February 8, 1900.

**Published:** 1900-05

**Authors:** Mazyck P. Ravenel

**Affiliations:** Bacteriologist to the State Live-Stock Sanitary Board of Pennsylvania; Lecturer on Bacteriology, Veterinary Department, University of Pennsylvania


### AN EXPERIMENT IN THE TRANSMISSION OF SYPHILIS TO CALVES.[1]

By Mazyck P. Ravenel, M.D.,

BACTERIOLOGIST TO THE STATE LIVE-STOCK SANITARY BOARD OF PENNSYLVANIA; LECTURER ON BACTERIOLOGY, VETERINARY DEPARTMENT, UNIVERSITY OF PENNSYLVANIA.

(*From the Laboratory of the State Live-stock Sanitary Board.*)

That syphilis is a disease peculiar to man and incapable of transmission to the lower animals is generally accepted by the medical profession, and upon such firm ground does this opinion appear to stand that most text-books make the statement without going into the details of the experiments on which it is based. Apart from the scientific interest which the subject bears, there is a prac-

[1] Read before the Pathological Society of Philadelphia, February 8, 1900.

tical importance attaching to it which must hold our attention, owing to the fact that the opponents of vaccination against smallpox lay great stress on the alleged danger of transmitting syphilis by vaccination, some of them even going so far as to claim that syphilis and vaccinia are one and the same disease. The agitation against vaccination in our own city and State during the year just closed, and the assertions made by certain doctors before a legislative committee of the dangers involved in the practice, must be fresh in the minds of all. It was this agitation that suggested to Dr. Leonard Pearson and myself the advisability of repeating once more the attempt to produce syphilis in calves by inoculation from man.

In looking over the record of experiments on this subject I have been unable to find any report of the successful inoculation of bovines, failure having resulted in every instance of which I have knowledge, though there are experimenters who have claimed success in inoculating rabbits, guinea-pigs, cats, dogs, horses, pigs, and monkeys—a fact which is somewhat significant.

The scope of this paper does not permit me to go into a critical review of past experiments, nor, indeed, do I think it necessary. I have been impressed, however, by the fact that several of those who have claimed success have employed material from soft chancres, while others have used tuberculous matter, as suggested by Striker, who examined the supposed syphilitic nodules induced by Hansell and also by Kaposi in regard to the same work.

The admirable work of Dr. Ernesto Duplan, done in the pathological laboratory of the University of Pennsylvania, and detailed in his graduation thesis (1884), deserves mention. He made seventy inoculations, using fifteen rabbits, five dogs, four guinea-pigs, and four apes, which he imported from Mexico himself for the purpose. His results were all negative, with the exception of two rabbits inoculated with pus from a soft chancre. In these an indurated lump formed at the point of inoculation, which opened spontaneously on the twelfth and thirteenth days respectively, discharging thick, cheesy pus. With this pus four rabbits were inoculated, one of which died of tuberculosis at the end of four weeks, the others showing no ill effects. Although he cites these two cases as examples of successful transmissions of syphilis to rabbits, I think it is clear that he was dealing with tuberculosis.

Among the lower animals there is only one disease known that bears any resemblance to syphilis—dourine or maladie du coit—sometimes called also syphilis of solipedes. This remarkable and interesting disease has been known for more than a hundred years, and is supposed by some to have had its origin in the coitus of syphilitic Arabs with she-asses. There is, however, absolutely no evidence of this, it being wholly theory, while on the contrary all attempts to produce dourine in mares and she-asses by infection with syphilitic virus have failed completely. Furthermore, the specifics against syphilis—mercury and iodide of potash—have no effect in dourine. The disease has been seen only twice in the United States, both times in imported stallions. It has been carefully studied in Europe, notably by the able veterinarians of the Alfort school, so that it is possible to affirm with certainty that dourine and syphilis are two entirely distinct diseases.

For our experiment two calves were selected, one a heifer, about eight months old, the other a bull, about fourteen months old, both in good condition, though the heifer was known to be tuberculous. Both had been on our premises and under observation for some months. The method of inoculation chosen was that practised by some of our best vaccine producers. The area selected was shaved, washed thoroughly with soap and water, and well rinsed with sterile water, after which a surface about the size of a silver half-dollar was scraped with a sharp scalpel until a slightly bloody serum began to exude. It was next scarified with the ordinary scarifier used in vaccination, in two directions at right angles to each other, and into this the syphilitic material was rubbed for not less than five minutes. A second scarification was then done, and the animals kept down until the surface had dried. The heifer (Ref. No. 26107) was inoculated in two places, on the inner side of the hind leg high up and on the abdomen just anterior to the udder, while the bull (Ref. No. 26130) was inoculated only on the inner side of the hind leg.

The material used was obtained through the kindness of a physician from a patient in the venereal ward of a hospital with which he is connected, and consisted of scrapings from a mucous patch on the lip and a sore about the genitals. The case was in the early secondary stage and the material taken at the commencement of treatment. It was collected at 11.30 A.M., and the inoculations made at 2 P.M. the same day.

On the next day the scarified areas were covered with a thin scab, and were surrounded by a narrow zone of slight redness, which disappeared during the next thirty-six hours. By the fifth day the scabs had loosened at the edges and dropped off on the ninth day. At no time was there any appreciable amount of inflammation or swelling, nor did the animals show any discomfort. The temperatures were taken soon after the inoculation, and twice daily for fifteen days, as given below:

RECORD OF TEMPERATURE.

| Calf No. 26107. | | | Calf No. 26130. | | |
|---|---|---|---|---|---|
| Date. | A. M. | P. M. | Date. | A. M. | P. M. |
| August 12 . . . | . . . . | 102.6 | August 12 . . . | . . . . | 103.6 |
| " 13 . . . | 102.2 | 104.2 | " 13 . . . | 102.6 | 104.2 |
| " 14 . . . | 102 | 102.3 | " 14 . . . | 102.8 | 102.2 |
| " 15 . . . | 102.4 | 102.6 | " 15 . . . | 101.6 | 101 |
| " 16 . . . | 102 | 102 | " 16 . . . | 102.4 | 102 |
| " 17 . . . | 101 | 102 | " 17 . . . | 101.2 | 102.4 |
| " 18 . . . | 101.2 | 101 2 | " 18 . . . | 102 | 101.4 |
| " 19 . . . | 101.3 | 102 | " 19 . . . | 101.2 | 101.2 |
| " 20 . . . | 101 | 102 | " 20 . . . | 102.2 | 102.2 |
| " 21 . . . | 101.2 | 102 | " 21 . . . | 102 | 102 |
| " 22 . . . | 102 | 102 | " 22 . . . | 102 | 102.2 |
| " 23 . . . | 102 | 102 | " 23 . . . | 101.8 | 102 |
| " 24 . . . | 101.4 | 101.8 | " 24 . . . | 102 | 102 |
| " 25 . . . | 101.6 | 102 | " 25 . . . | 101.4 | 101.8 |
| " 26 . . . | 102 | 101.8 | " 26 . . . | 101.6 | 101.4 |
| " 27 . . . | 102 | 102 | " 27 . . . | 101.6 | 102 |

Calf No. 26107 was kept under observation until October 5th, fifty-four days after inoculation, when it was killed. It was impossible to detect the points of inoculation, nor could any lesions be found in any part of the body which were in the least suggestive of syphilis. The animal was known to have tuberculosis, which the examination showed to be very limited, confined to a few nodules in the lung. A portion of the sciatic nerve, taken from the region of inoculation, and a section of the dorsal spinal cord were sent to the Pepper Clinical Laboratory, where they were examined by Dr. McCarthy, whose report is as follows:

"The examination of the central nervous system and the peripheral nerves of the calf No. 26107, inoculated with syphilis and sent to me by you, revealed no changes either by the Marchi osmic acid method, the Nissl cell stain, or the Weigert sheath stain. The peripheral nerves teased and stained by osmic acid were likewise normal. D. J. McCarthy."

Calf No. 26130 was kept under observation until December 28th, one hundred and thirty-eight days, when it was killed. A few days before it had been tested with tuberculin and found to react. In this animal, as in the other, it was impossible to detect the point of inoculation, nor could any change in the skin be detected after removal. The organs were entirely normal with the exception of one lung, which contained a cheesy tubercular nodule three-fourths of an inch in diameter. Dr. McCarthy's report on the nerves and spinal cord is as follows:

"An examination of the central nervous system and peripheral nerves of calf No. 26130, inoculated with syphilis, gave negative results with the fresh osmic acid method, Marchi method, Weigert and Nissl stains. The specimens were perfectly normal.

"D. J. McCarthy."

The result of these two experiments, which were conducted with the greatest care, has been confirmatory of the work of those observers who, like Neumann, have failed in all attempts to transmit syphilis to the lower animals, and who hold it to be impossible.

I apprehend that some may object to the value of these experiments, on the ground that the animals were both tuberculous, a fact which did not escape my attention. In man the two diseases are not rarely met with coexisting in the same individual, and the presence of tuberculosis appears to make the patient more susceptible to syphilis. Says Landouzy: "In this association syphilis finds the subject without resistance, and, furthermore, affording no hold to specific medication . . . The worst morbid association that I know is the union of a pulmonary tuberculosis with a commencing syphilis" (*Congrès pour l'étude de la Tuberculose*, 1891).

In closing, I wish to make acknowledgment of the efficient services of my assistant, Mr. S. H. Gilliland, in the carrying out of this experiment, and of my indebtedness to Dr. D. J. McCarthy for his examination of the nerves and spinal cord.

